# The diversity of AMPA receptor inhibition mechanisms among amidine-containing compounds

**DOI:** 10.3389/fphar.2024.1467266

**Published:** 2024-10-09

**Authors:** Arseniy S. Zhigulin, Mikhail Y. Dron, Oleg I. Barygin, Denis B. Tikhonov

**Affiliations:** Laboratory for the Research of the Mechanisms of Regulation and Compensation of Nervous System Excitability Pathologies, I.M. Sechenov Institute of Evolutionary Physiology and Biochemistry RAS, Saint Petersburg, Russia

**Keywords:** amidine compounds, AMPA receptor, inhibition mechanisms, patch-clamp technique, pharmacological modulation

## Abstract

Amidine-containing compounds are primarily known as antiprotozoal agents (pentamidine, diminazene, furamidine) or as serine protease inhibitors (nafamostat, sepimostat, camostat, gabexate). DAPI is widely recognized as a fluorescent DNA stain. Recently, it has been shown that these compounds also act as NMDA receptor inhibitors. In this study, we examined the activity of these compounds and analyzed the mechanisms of action in relation to another important class of ionotropic glutamate receptors–calcium-permeable AMPA receptors (CP-AMPARs) and calcium-impermeable AMPA receptors (CI-AMPARs) – using the whole-cell patch-clamp method on isolated male Wistar rat brain neurons. Gabexate and camostat were found to be inactive. Other compounds preferentially inhibited calcium-permeable AMPA receptors with IC_50_ values of 30–60 µM. DAPI and furamidine were also active against CI-AMPARs with IC_50_s of 50–60 μM, while others showed poor activity. All active compounds acted as channel blockers, which are able for permeating into the cytoplasm on both CP- and CI-AMPARs. Specifically, sepimostat showed trapping in the closed CP-AMPAR channel. Furamidine and DAPI demonstrated a voltage-independent action on CI-AMPARs, indicating binding to an additional superficial site. While the majority of compounds inhibited glutamate-activated steady-state currents as well as kainate-activated currents on CI-AMPARs, pentamidine significantly potentiated glutamate-induced steady-state responses. The potentiating effect of pentamidine resembles the action of the positive allosteric modulator cyclothiazide although the exact binding site remains unclear. Thus, this study, together with our previous research on NMDA receptors, provides a comprehensive overview of this novel group of ionotropic glutamate receptors inhibitors with a complex pharmacological profile, remarkable diversity of effects and mechanisms of action.

## 1 Introduction

Glutamate is the primary excitatory neurotransmitter in the central nervous system, acting through different glutamate receptors. Among these receptors are ionotropic glutamate receptors of the NMDA and AMPA types, which are crucial for the generation of excitatory postsynaptic currents (Hansen et al., 2021). AMPA receptors are typically categorized into two subtypes: calcium-permeable (CP-AMPARs) and calcium-impermeable (CI-AMPARs), each distinguished by their unique biophysical and pharmacological properties. Specifically, CP-AMPARs exhibit higher sensitivity to cationic channel blockers (Magazanik et al., 1997; Mellor and Usherwood, 2004) and lower sensitivity to the anticonvulsant phenytoin (Dron et al., 2021). Interestingly, both CP-AMPARs and CI-AMPARs display similar sensitivity to the allosteric antagonist perampanel (Barygin, 2016).

In conditions of overstimulation, excessive calcium influx through NMDA and calcium-permeable AMPA receptors leads to excitotoxic effects, contributing to the pathogenesis of various neurodegenerative diseases (Weiss, 2011). The current list of inhibitors targeting NMDA and AMPA receptors used in clinics is short: it includes NMDA receptor channel blockers such as memantine, ketamine, and dextromethorphan, as well as the AMPA receptor allosteric antagonist perampanel. Memantine is used in the treatment of Alzheimer’s disease (Lipton, 2006). Ketamine serves as a dissociative anesthetic and rapid-acting antidepressant (Berman et al., 2000; Chaki and Watanabe, 2023). Dextromethorphan is an antitussive agent recently approved for the treatment of major depressive disorder in combination with bupropion (Keam, 2022). Perampanel is used for the treatment of partial-onset seizures and generalized tonic-clonic seizures (Rogawski and Hanada, 2013; Patsalos, 2015). Therefore, the search for new inhibitors of ionotropic glutamate receptors is currently of great interest. The favorable clinical profile of memantine is attributed to its moderate affinity, rapid kinetics, and partial trapping (Lipton, 2006). In contrast, many other NMDA receptor channel blockers with higher affinity and slower kinetics cannot be used in clinical practice due to severe side effects (Rogawski and Wenk, 2003; Kalia et al., 2008). In the same time the advantage of perampanel lies in its specific non-competitive allosteric mechanism of AMPA receptor negative modulation (Hanada et al., 2011; Yelshanskaya et al., 2016; Yuan et al., 2019). Thus, it is essential to consider not only the possibility of inhibition and activity but also the mechanism of action during the development of new drugs.

The study of compounds that are already used as pharmacological agents can significantly save time and expenses on clinical safety studies. Amidine-containing compounds are examples of such pharmacological agents. Pentamidine is an anti-infective diarylamidine compound used to treat African trypanosomiasis, leishmaniasis, and prevent/treat pneumocystis pneumonia in immunocompromised individuals. It binds to the DNA minor groove (Baraldi et al., 2004) and intercalates into RNA (Jarak et al., 2011). Additionally, it exhibits neuroprotective properties *in vitro* as an NMDA receptor inhibitor (Reynolds and Aizenman, 1992). Diminazene, also known as berenil, is another anti-infective diarylamidine medication primarily used in animals to treat trypanosomiasis due to serious side effects preventing its use in humans. Aside from DNA and RNA intercalation, diminazene also interacts with various enzymes including angiotensin-converting enzyme (Pilch et al., 1995; da Silva Oliveira and de Freitas, 2015). Studies have shown it to induce peripheral antihyperalgesia in a rat model of chronic inflammatory pain and possess immunomodulatory properties affecting crucial signaling pathways associated with cytokine production (Kuriakose and Uzonna, 2014; Lee et al., 2018). 4′,6-diamidino-2-phenylindole (DAPI) is a diarylamidine compound widely recognized as a fluorescent DNA stain but not used as a medication (Larsen et al., 1989). Furamidine, a pentamidine analogue, demonstrates antiparasitic properties (Purfield et al., 2009). Nafamostat, a serine protease inhibitor developed in Japan initially for acute pancreatitis treatment (Iwaki et al., 1986), is also employed as an anticoagulant (Akizawa et al., 1993). Sepimostat, a structurally related protease inhibitor to nafamostat, possesses enhanced oral bioavailability compared to nafamostat but was discontinued for clinical use for unknown reasons. In addition, it has been shown that both nafamostat and sepimostat have retinoprotective properties (Fuwa et al., 2019). It's worth mentioning that nafamostat, sepimostat, pentamidine, diminazene, and DAPI are blockers of acid-sensing ion channels (Ugawa et al., 2007; Chen et al., 2010; Schmidt et al., 2017; Zhigulin et al., 2023). Gabexate and camostat are clinically used serine protease inhibitors (Nakahara, 1983; Yin et al., 2005).

In our recent papers, we performed a systematic analysis of amidine-containing compounds action against NMDA receptors in rat brain neurons (Dron et al., 2020; Zhigulin and Barygin, 2022; Zhigulin and Barygin, 2023). Except for camostat, which was ineffective, these compounds demonstrated activities in the range from 0.2 to 16 µM and were shown to have two binding sites on NMDA receptors. They bound to the superficial site with similar affinity, the differences in the blocking action were due to different binding to the channel site.

In another study, we demonstrated that diminazene also inhibits both calcium-permeable and calcium-impermeable AMPA receptors (Zhigulin et al., 2022). It acted as a permeable open-channel blocker and was more active against CP-AMPARs. To the best of our knowledge, other compounds of this structural family have not been thoroughly tested for activity against CP- and CI-AMPAR subtypes, and we decided to fill this gap in the present work.

The analysis of structurally related compounds allows for a better understanding of structure-activity relationships and mechanisms of action. Chemical structures of amidine-containing compounds studied are presented in Supplementary Table S1. Diarylamidine compounds—pentamidine, diminazene, DAPI, and furamidine—contain two positively charged at physiological pH amidine groups, connected by different linkers. Nafamostat is structurally similar to diarylamidine compounds but contains one guanidine group and one amidine group, both of which are positively charged. Sepimostat is a monocationic analog of nafamostat, containing an uncharged 4,5-dihydro-1H-imidazole-2-ylamino group instead of a guanidine group. Gabexate and camostat are monovalent cations with one guanidine group, but their 3D structures differ significantly from those of nafamostat and sepimostat. Considering these structural differences and the complexity of NMDA receptor inhibition mechanisms among these compounds, we suspected that they could also affect AMPA receptors in different ways. In this study, we conducted an analysis of AMPA receptor inhibition by amidine-containing compounds and demonstrated the diversity of their mechanisms of action.

## 2 Methods

### 2.1 Animals

All experimental procedures were approved by the Animal Care and Use Committee of the Sechenov Institute of Evolutionary Physiology and Biochemistry of the Russian Academy of Sciences (protocol 1-18/2022, 27 January 2022). Outbred male Wistar rats (13–18 days old and weighing 25–35 g) were obtained from a local (IEPHB) facility. Maximum efforts were made to minimize the number of animals used and to minimize discomfort.

### 2.2 Electrophysiology

The rats were anesthetised with sevoflurane and then decapitated. The brains were brought out quickly and cooled to 2°C–4°C. Transverse striatal or hippocampal slices were cut using a vibratome (Campden Instruments) and stored in a solution containing (in mM): NaCl 124, KCl 5, CaCl_2_ 1.3, MgCl_2_ 2.0, NaHCO_3_ 26, NaH_2_PO_4_ 1.24, D-glucose 10, aerated with carbogen (95% O_2_, 5% CO_2_). All experiments were performed at room temperature.

Vibrodissociation method (Vorobjev, 1991; Jun et al., 2011) was used to free CA1 pyramidal neurons or giant striatal interneurons from slices. This method allows isolating cells without enzymatic treatment and keep them in more native state. The antagonism of CP-AMPARs was studied on striatal giant interneurons (Bernard et al., 1997; Gotz et al., 1997), which were identified by their shape and size. They have large (>25 µm) soma of polygonal shape, whereas principal cells are significantly smaller and nearly spherical. Previous works demonstrated that a non-desensitizing response to kainate in these neurons is mediated by GluA2-lacking AMPARs (Samoilova et al., 1999). The sensitivity to dicationic blockers like IEM-1460, IEM-1925, and polycationic toxins agrees with the data on recombinant receptors (Bolshakov et al., 2005; Barygin et al., 2011). The currents demonstrate inward rectification and significant Ca^2+^ permeability (Buldakova et al., 1999; Samoilova et al., 1999). The antagonism of CI-AMPARs was studied on pyramidal neurons from the CA1 area of the hippocampus. These cells were isolated from the stratum pyramidale and distinguished from non-pyramidal cells on the basis of pyramidal-like somata and preserved apical dendrites. Kainate-induced currents in these neurons are virtually insensitive to cationic blockers (Magazanik et al., 1997; Bolshakov et al., 2005) but are sensitive to phenytoin (Dron et al., 2021).

To record membrane currents in response to applications of kainate or glutamate the whole-cell configuration of patch clamp technique was used. Series resistance (<20 MΩ) was compensated by 70%–80% and monitored during experiments. Only cells with stable holding currents were used in further analysis. The current signals were amplified using EPC-8 (HEKA Electronics), filtered at 5 kHz, sampled and stored on a personal computer. RSC-200 (BioLogic) perfusion system was used to apply the drugs under computer control. The solution exchange time in the whole-cell mode was about 200 ms. The composition of extracellular solution (in mM) was: NaCl 143, KCl 5, CaCl_2_ 2.5, MgSO_4_ 2, D-glucose 18, HEPES 10 (pH adjusted to 7.4 with HCl). The pipettes with resistance of 2–5 MΩ were filled with the following solution (in mM): CsF 100, CsCl 40, NaCl 5, CaCl_2_ 0.5, EGTA 5, HEPES 10 (pH adjusted to 7.2 with CsOH). Pentamidine isethionate (P-155) was from Alomone laboratories. Other reagents were purchased from MedChemExpress (Monmouth Junction, NJ, United States), Sigma (St. Louis, MO, United States) or Tocris Bioscience (Bristol, UK).

AMPA receptors were activated with 100 µM kainate or 1 mM glutamate unless otherwise stated. In case of activation by glutamate we used D-AP5 (100 µM) for full exclusion of NMDA receptors activation. The effects of compounds on steady-state currents for different drug concentrations were measured at −80 mV holding voltages. The inhibitory effects are shown as percentages of inhibition (100%–100% I_drug_/I_control_). In the case of complex effects of inhibition and potentiation, the total effect is shown as I_drug_/I_control_ ratio. Kinetics of transient processes of more than 20 ms duration were approximated by single or double exponential functions. In case of double exponential fitting, the weighted time constant was used.

### 2.3 Analysis of voltage dependence

The voltage dependence of compounds action was analysed by Woodhull model for permeable blockers (Woodhull, 1973; Tikhonova et al., 2008). According to this model, the voltage dependence of steady-state blockade is given by Equation 1:

$$B\left(V\right)=100\%-\frac{100\%}{1+\frac{C}{K_{b}\exp\left(\frac{F}{RT\;}z\delta_{b}\;V\right)+K_{p}\;\exp\left(-\frac{F}{RT\;}z\delta_{p}\;V\right)}}$$ (1)

where V is voltage, B is level of block (%), C is concentration of the drug, z is molecular charge, and R, F and T have their standard meanings. K_b_ is the affinity of a drug for the channel. The δ_b_ value reflects the fraction of membrane electric field that the charged blocking molecule crosses on its pathway between the external media and the binding site in the channel (where the total field has a value of 1). Parameters K_p_ and δ_p_ describe permeation through the channel. In some cases, the data for CI-AMPARs could not be well fitted with Equation 1 due to pronounced inhibition at positive voltages, which suggests the presence of voltage-independent component of action. For this reason, we used Equation 2, which takes into account the possibility of inhibitor to bind also at superficial site (Nikolaev and Tikhonov, 2023), assuming that the binding to the deep and superficial site is independent.

$$B\left(V\right)=100\%-\frac{100\%}{1+\frac{C}{K_{b}\exp\left(\frac{F}{RT\;}z\delta_{b}\;V\right)+K_{p}\;\exp\left(-\frac{F}{RT\;}z\delta_{p}\;V\right)}+\frac{C}{K_{vin}}+\frac{C^{2}}{K_{vin}\;\left(K_{b}\exp\left(\frac{F}{RT\;}z\delta_{b}\;V\right)+K_{p}\;\exp\left(-\frac{F}{RT\;}z\delta_{p}\;V\right)\right)}}$$ (2)

In this equation K_vin_ is the affinity of the drug to the superficial site and other parameters are the same as in Equation 1.

### 2.4 Statistical analysis

All experimental data are presented as the mean ± SD estimated from at least four experiments (cells). Significance of the effects was tested with t-tests. Differences were considered significant at *p* < 0.05. Concentration dependencies were approximated by Hill equation. Voltage dependencies were approximated by Equation 1 or 2. Patch destabilization with a large number of transitions between different potential states did not allow an estimation of the entire voltage dependence in a single experiment, and the inhibitory effects at different holding voltages were estimated in independent experiments. Then all data was pooled together and the parameters of voltage dependence were estimated using the fitting of all measurements. For each holding potential ≥4 cells were used. Voltage dependence parameters (binding constants and δ_b_ values) are shown as the result of the fitting ± approximation errors.

### 2.5 Molecular modeling

Molecular modeling was performed using ZMM program package as described previously (Barygin et al., 2011). The nonbonded energy was calculated using the AMBER force field (Weiner et al., 1986), and the hydration energy was calculated using the implicit solvent method (Lazaridis and Karplus, 1999). Electrostatic interactions were calculated using the distance-dependent dielectric function, and the atomic charges of compounds were calculated by the semiempirical method AM1 (Dewar et al., 1985). The Monte Carlo with energy minimizations method (Li and Scheraga, 1987) was used to optimize the models and their complexes with drugs. During energy minimizations, alpha carbons of the P-helices were constrained to corresponding positions of the template using constraints. The models were optimized until 1,000 consecutive minimizations did not decrease the energy of the apparent global minimum.

## 3 Results

### 3.1 Screening for activity and concentration dependence

**FIGURE 1:**
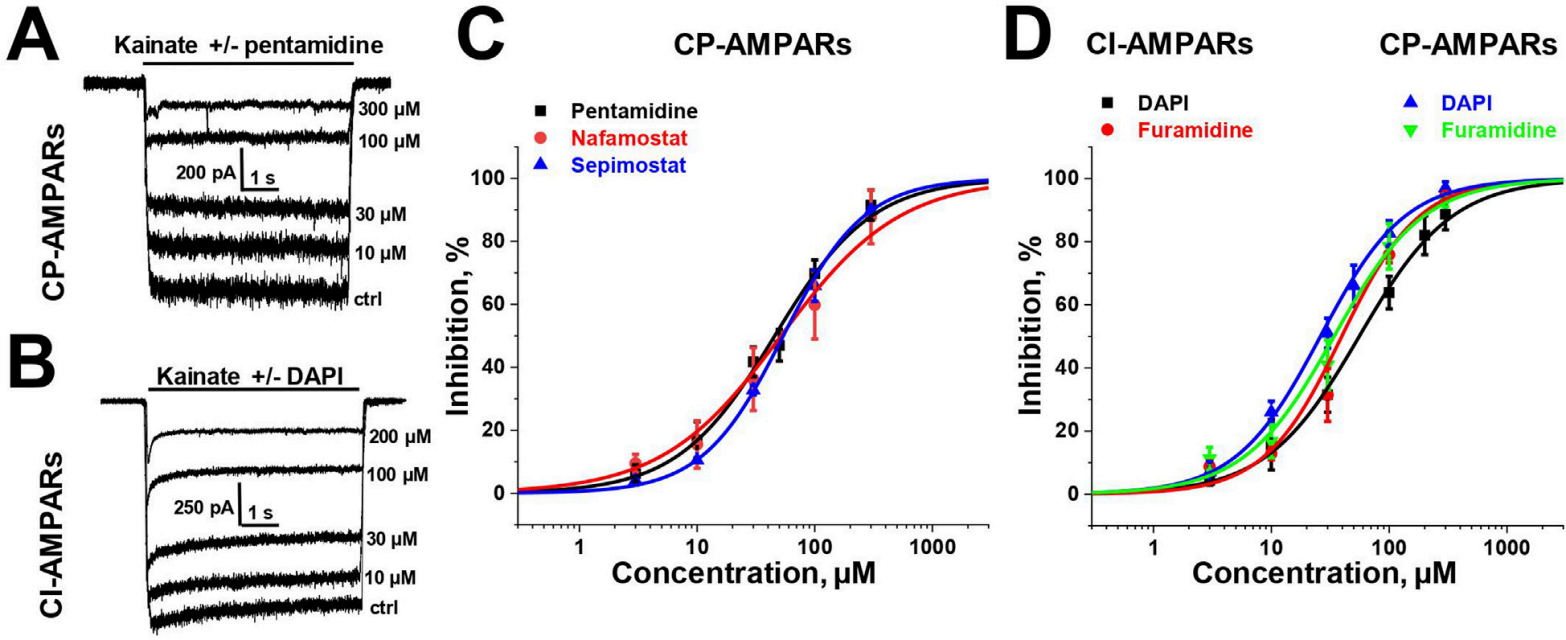
Concentration dependencies of active amidine-containing compounds action on CP- and CI-AMPARs. **(A, B)** Representative examples of kainate-induced currents inhibition by different concentrations of pentamidine on CP-AMPARs **(A)** and DAPI on CI-AMPARs **(B)** **(C, D)** Concentration-inhibition curves for active compounds.

To estimate the activity of amidine-containing compounds against calcium-permeable and calcium-impermeable AMPA receptors, we measured the percentage of kainate-induced current block by each compound at a holding voltage of −80 mV and a concentration of 100 µM. Striatal giant interneurons and hippocampal pyramidal cells (CA1 area) were used for CP-AMPARs and CI-AMPARs, respectively. Results are presented in Supplementary Table S1. Gabexate and camostat were found to be inactive (block <50% for both types of AMPARs). Except for furamidine, which demonstrated nearly the same activity against both types of AMPARs (∼75–80% block), all compounds preferably inhibited calcium-permeable AMPA receptors, showing approximately 60%–80% block for CP-AMPARs and 40%–60% block for CI-AMPARs. In cases where the block was over 50%, we applied different concentrations of compounds and estimated the IC_50_ values (Supplementary Table S1; Figure 1). The IC_50_ values for CP-AMPARs were in the range of 30–60 µM: DAPI, furamidine, and pentamidine were more active with IC_50_s of 30–40 μM, whereas nafamostat and sepimostat demonstrated similar activity at around 55 μM, which is close to that of the previously studied diminazene (Zhigulin et al., 2022). DAPI and furamidine were also active against CI-AMPARs with IC_50_s of 50–60 µM. The Hill coefficients for all concentration dependencies were in the range of 1.0–1.4, suggesting the absence of cooperativity effects.

### 3.2 Voltage dependence

**FIGURE 2:**
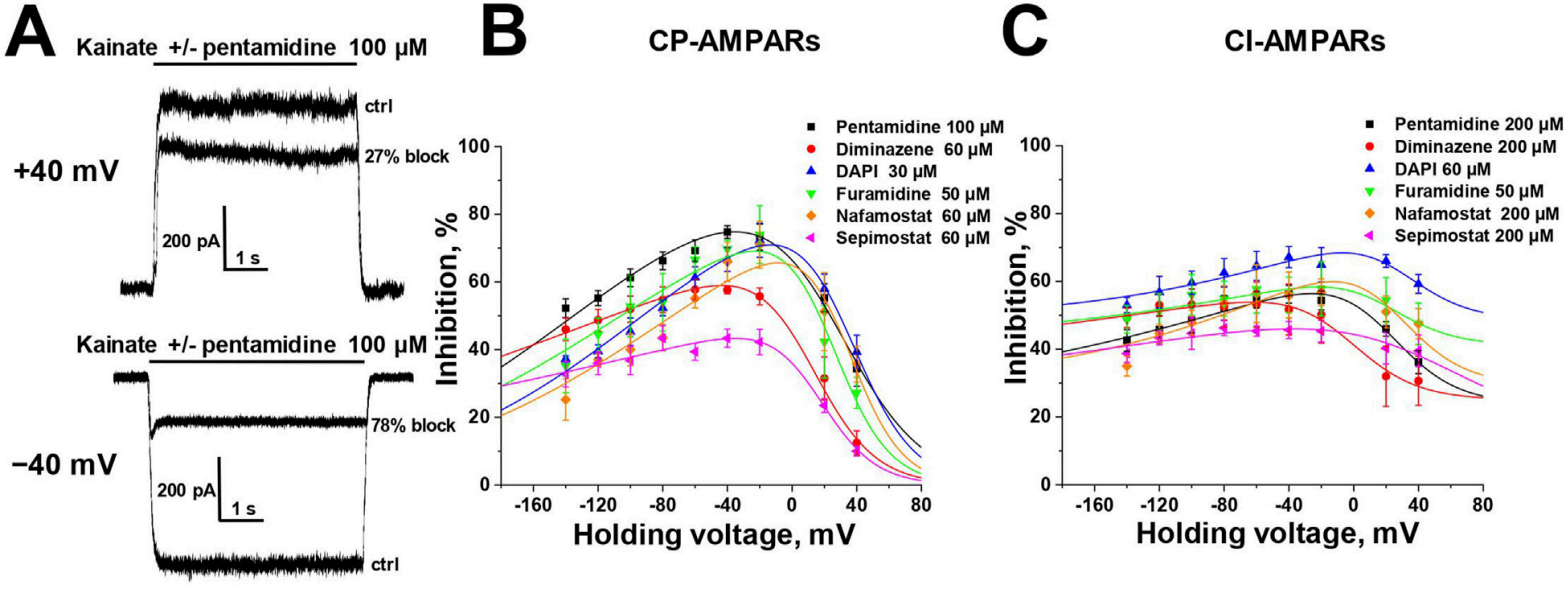
Voltage dependence of amidine-containing compounds action on CP- and CI-AMPARs. **(A)** Representative examples of CP-AMPARs block by 100 µM pentamidine at different holding voltages. **(B, C)** The voltage dependence data for CP-AMPARs inhibition by amidine-containing compounds **(B)** was fitted by Equation 1, whereas the data for CI-AMPARs inhibition **(C)** was fitted by Equation 2.

Selective blockade of CP-AMPARs is a classical feature of the action of positively charged open-channel blockers (Magazanik et al., 1997). To reveal the pore blocking action of the amidine-containing compounds, we measured the inhibitory effect of a fixed concentration (that caused ∼50% block at −80 mV) of each active compound at different holding voltages within the range of −140 to +40 mV (see Figure 2) for both CP- and CI-AMPARs.

In the case of CP-AMPARs, the voltage dependencies of all tested compounds exhibited a pronounced bell-shaped form with the maximal inhibition at −40 to 0 mV (Figure 2B). For impermeable blockers, the voltage dependence shows monotonic increase in inhibition with hyperpolarization, as in the case of NMDARs (Zhigulin and Barygin, 2023). The bell-shaped voltage dependence is a characteristic feature of channel blockers that can permeate through the channel into the cytoplasm (Tikhonova et al., 2008). At high negative voltages, the strong electric field forces charged molecule to permeate, resulting in a reduction of inhibition with hyperpolarization. Therefore we fitted the voltage-dependence data using the Woodhull model for permeable blockers (Woodhull, 1973; Tikhonova et al., 2008) (Equation 1, see Methods).

The δ_b_ values presented in Supplementary Table S2, serving as indicators of the membrane electric field fraction that the charged blocking molecule crosses on its pathway between the external media and the binding site in the channel (Tikhonov and Magazanik, 1998), demonstrate a close similarity for dicationic diminazene, DAPI, furamidine, and nafamostat (around 0.7) with our earlier data on the dicationic derivatives of adamantane and phenylcyclogexyl (Tikhonova et al., 2008). The δ_b_ value for dicationic pentamidine was found to be the lowest at ∼ 0.55, while the δ_b_ value for monocationic sepimostat was ∼0.9. In the case of dicationic compounds that bind in the pore in an axial orientation, the δ_b_ value corresponds to a position between the charged groups. The higher δ_b_ value for monocationic sepimostat suggests that it binds in an orientation where the charged guanidine group is located deep in the channel pore, while the uncharged group at the opposite end occupies a more superficial position.

In the case of CI-AMPARs, the bell-shaped voltage dependencies were not as pronounced as those observed for CP-AMPARs. This suggests the presence of a voltage-independent component of action, which obscures the components responsible for voltage-dependent block (Figure 2C). Consequently, describing these data using a model solely based on binding in the channel pore is impractical. We employed Equation 2 (see Methods), which also considers potential voltage-independent binding (Nikolaev and Tikhonov, 2023). The K_vin_ value in Equation 2 describes the affinity to a superficial site. Due to the increased complexity of Equation 2 compared to Equation 1, it was impossible to estimate all parameters in case of CI-AMPARs reliably. However, the data were well fitted using fixed δ_b_ values obtained from the voltage dependence data analysis on CP-AMPARs. The parameters of the voltage dependencies of CP- and CI-AMPARs block are shown in Supplementary Table S2.

For all compounds, the K_b_ values, which reflect the binding affinity to the site in the pore, were lower for CP-AMPARs than for CI-AMPARs, suggesting preferable binding to the CP-AMPARs as in the case of other cationic compounds. DAPI and nafamostat demonstrated the highest affinity at both CP- and CI-AMPAR channels, with binding constants of 5–10 µM for CP-AMPARs and 25–80 µM for CI-AMPARs. Other compounds had K_b_ values in the range of 15–70 µM for CP-AMPARs and >100 µM for CI-AMPARs. It is worth noting that for furamidine, which showed a poor difference in activity against CP- and CI-AMPARs at −80 mV holding voltage (IC_50_ values of 38 and 47 μM, respectively), the analysis of voltage dependence revealed a significant difference in K_b_ values (15 and 110 μM, respectively). This underscores the importance of analyzing voltage dependence parameters for accurate affinity estimation. Pentamidine, diminazene, nafamostat, and sepimostat acted with relatively weak affinity for the superficial site on CI-AMPARs (K_vin_ > 400 µM). In contrast, both furamidine and DAPI, which were active at −80 mV holding voltage (IC_50_ < 100 µM) on CI-AMPARs, exhibited pronounced voltage-independent action (K_vin_ < 100 µM). This explains the high activity of furamidine and DAPI on CI-AMPARs at −80 mV, as it is significantly influenced by their action at the superficial site.

### 3.3 Mechanisms of CP-AMPAR channel block by sepimostat and pentamidine

**FIGURE 3:**
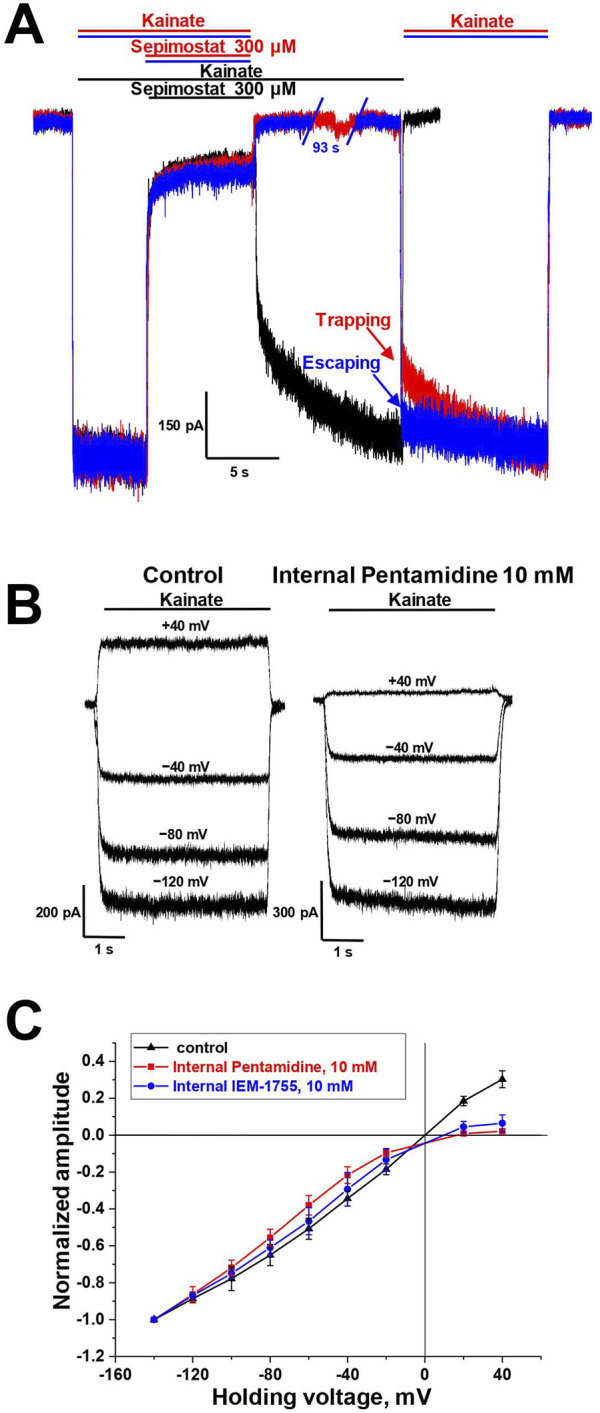
Mechanisms of CP-AMPAR channel block by sepimostat and pentamidine. **(A)** Trapping of 300 µM sepimostat on CP-AMPARs. Sepimostat demonstrates slow washout kinetics (black trace). Sepimostat demonstrates trapping after 10 s pause in extracellular solution (red trace) and escapes from trapping after 100 s pause (blue trace). **(B, C)** Internal block of open CP-AMPAR channel by 10 mM pentamidine and 10 mM IEM-1755. **(B)** Representative examples of kainate-induced currents at different holding voltages in control and with internal pentamidine. **(C)** I-V relation represents the inhibition effect by internally applied pentamidine (red) and IEM-1755 (blue) at low negative and positive voltages.

There are two major mechanisms of ion channels blockade: “foot-in-the-door” and trapping block (MacDonald et al., 1991; Vorobjev and Sharonova, 1994; Benveniste and Mayer, 1995; Blanpied et al., 1997; Sobolevsky, 1999). The blockers that bind to an open channel and are able to stay bound after agonist dissociation and channel closure are trapping blockers. In contrast, “foot-in-the-door” blockers interact with the channel gating mechanism and prevent channel closure, making it impossible for the channel to close before the blocker dissociates. For instance, “foot-in-the-door” and trapping channel blockers differentially affect synaptic activity with different frequencies (Zaitsev et al., 2011). The trapping mechanism of AMPAR channel block is well-characterized (Tikhonova et al., 2008). Among the compounds studied, only sepimostat demonstrated slow recovery kinetics (τ = 1.5 ± 0.3 s, n = 4), which allowed us to test its action in simple trapping protocol (Bolshakov et al., 2003) (Figure 3A). The full recovery from block by 300 µM sepimostat was achieved after 10 s in the presence of kainate (Figure 3A, black trace). In contrast, in the trapping protocol, after 10 s without kainate, the significant inhibition effect (21% ± 2%, n = 4) was observed (Figure 3A, red trace), indicating that recovery in the absence of kainate is slower than in its presence. We also performed this experiment with a longer (100 s) time interval and found that the inhibition effect had practically disappeared (Figure 3A, blue trace). These data fully agree with previous results (Tikhonova et al., 2008). Sepimostat, like other AMPAR channel blockers, gets trapped in the closed channel but can slowly escape from trapping by leaking into the cytoplasm. The fast recovery kinetics of other compounds prevented the direct estimation of the trapping effect, but the similarity of voltage dependencies suggests the same mode of action.

It was previously demonstrated that channel blockers capable of permeating through the channel into the cytoplasm are also active when applied internally (Tikhonova et al., 2009). For instance, spermine is an AMPAR channel blocker known to induce inward rectification (Bowie and Mayer, 1995; Kamboj et al., 1995; Koh et al., 1995). Therefore, we aimed to investigate whether the presence of 10 mM pentamidine in the pipette solution would affect the shape of the current-voltage relationship (I-V curve) for kainate-induced responses in CP-AMPA receptors. In these experiments, we used the classical blocker IEM-1755 (10 mM) as a reference compound. In the control condition, the I-V curve for kainate-induced responses was linear (Figures 3B, C). With the presence of pentamidine or IEM-1755 in the pipette solution, the curve demonstrated strong inward rectification (Figures 3B, C), indicating that both pentamidine and IEM-1755 have the ability to access their binding sites when applied internally. The rectification index (current ratio between +40 mV and −40 mV) was 0.9 ± 0.1 (n = 4) in control, 0.10 ± 0.04 (n = 4) for pentamidine, and 0.22 ± 0.05 (n = 4) for IEM-1755. Therefore, similar to other permeant blockers, pentamidine can block AMPA receptors channels from the intracellular side.

### 3.4 Pentamidine is more active in conditions favoring open AMPAR channels

Earlier it was shown that externally applied trapping blockers, which are able to permeate through the channel, are more effective in conditions favoring open channels (Zaitsev et al., 2011). This is because these blockers can escape from closed channels into the cytoplasm, while accumulation of the drug in the binding site is possible only when the channel is open.

**FIGURE 4:**
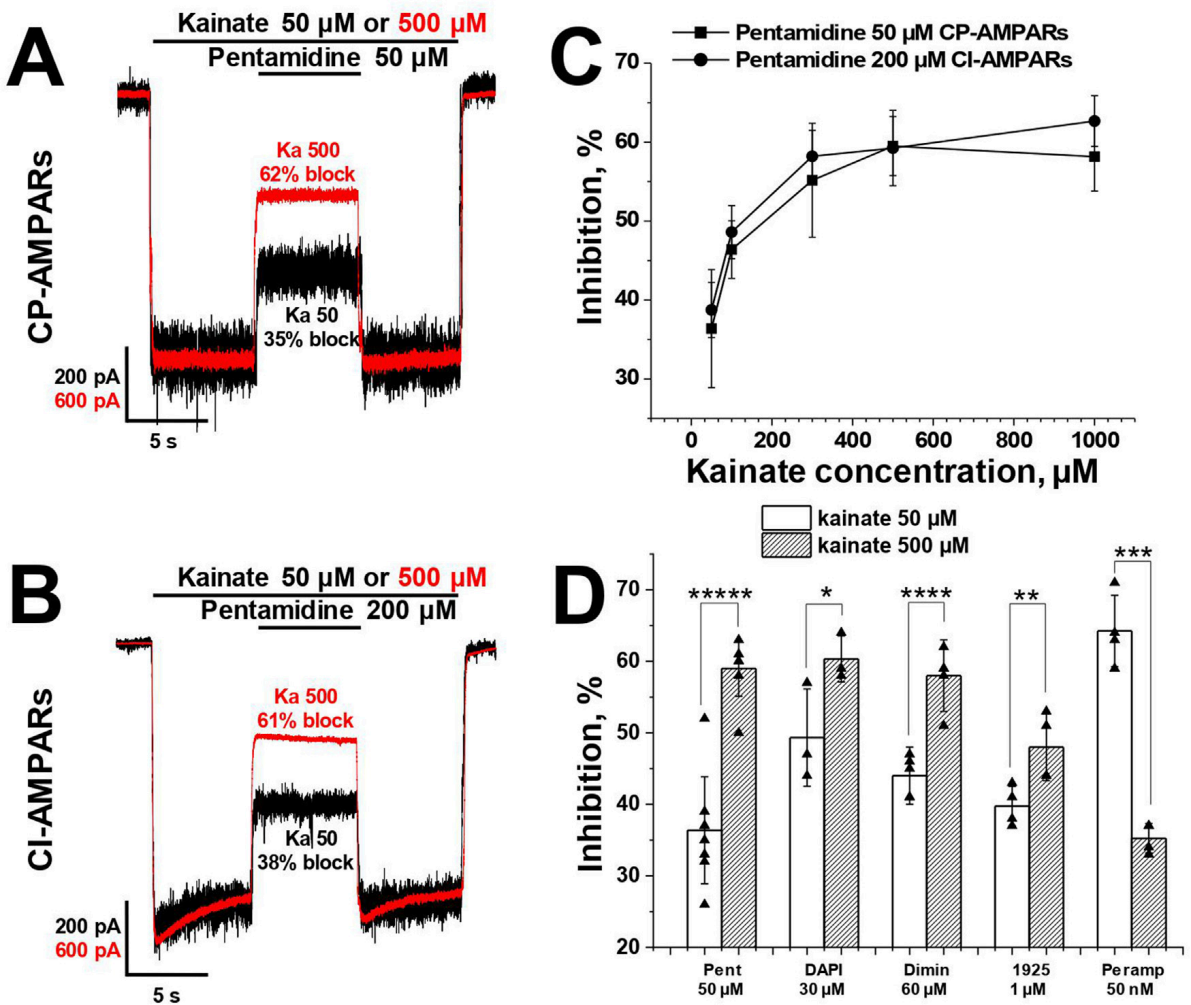
The activity of pentamidine at −80 mV holding voltage increases with an increase of kainate concentration. **(A, B)** Representative examples of pentamidine action in kainate 50 and 500 µM on CP- **(A)** and CI-AMPARs **(B)**. **(C)** The action of pentamidine at five different kainate concentrations on CP- and CI-AMPARs. **(D)** Summary of agonist dependence data for pentamidine 50 µM and DAPI 30 µM in comparison with previously studied diminazene 60 μM, IEM-1925 1 µM and perampanel 50 nM on CP-AMPARs. Paired t-test: * – *p* = 0.04 (n = 4), ** – *p* = 0.008 (n = 4), *** – *p* = 0.002 (n = 4), **** – *p* = 0.0009 (n = 4), ***** – *p* = 0.00002 (n = 5).

For the analysis of the agonist dependence of compounds action, we measured the inhibitory effect at −80 mV holding voltage during CP-AMPAR activation using low (50 μM, ∼10% of maximal response) and high (500 μM, ∼90% of maximal response) kainate concentrations. The classical pore blocker IEM-1925 (1 µM) caused 40% ± 3% inhibition with 50 µM kainate, and the inhibition increased to 48% ± 5% with 500 µM kainate (*p* = 0.008, n = 4, paired t-test). In contrast, the negative allosteric AMPAR antagonist perampanel (50 nM) was more effective at the 50 µM kainate concentration (*p* = 0.002, paired t-test, n = 4), consistent with previously published data (Dron et al., 2021). Pentamidine (50 μM, Figure 4A), DAPI (30 µM), and diminazene (60 µM) (Zhigulin et al., 2022) demonstrated a significant increase in the blocking effect with the increase in kainate concentration.

In the case of CI-AMPARs, pentamidine (200 µM) also demonstrated an increase in action with the increase in kainate concentration (Figure 4B). We also investigated the action of a single concentration of pentamidine at five different kainate concentrations (50–1,000 µM) on both CI- and CP-AMPARs (Figure 4C). In both cases, the percentage of inhibition by pentamidine increased monotonically with the increase in kainate concentration, reaching a plateau at 1,000 µM kainate. Summary of agonist dependence data on CP-AMPARs is represented in Figure 4D.

In contrast to pentamidine, the action of furamidine on CI-AMPARs is mostly determined by binding to a superficial site rather than the channel site (Supplementary Table S2). It showed the opposite trend, as in the case of kainate 500 µM furamidine 50 µM was slightly less active (48% ± 8% block, n = 7) than at 50 µM kainate (54% ± 6%, n = 7, *p* = 0.001, paired t-test). Therefore, the agonist dependence of the action was different for pore-blocking compounds (pentamidine, diminazene, DAPI) compared to compounds with predominantly voltage-independent action (furamidine on CI-AMPARs).

Taken together, the data presented above fully characterize the compounds used in the study as typical voltage-dependent pore blockers of CP-AMPARs, which become trapped in the closed channels when agonist is removed and can permeate through the pore into the cytoplasm. In the case of CI-AMPARs additional voltage-independent component of action was revealed. Note that the voltage-independent component of action on AMPARs was previously demonstrated for other compounds (Barygin et al., 2010).

### 3.5 Pentamidine potentiates and blocks glutamate-induced CI-AMPAR currents

Although kainate-induced AMPAR currents are convenient for investigating biophysical characteristics of the ligands action, there is a need to test compounds actions under conditions closer to physiological. Natural agonist glutamate and partial agonist kainate differently affect AMPAR desensitization (Vorobjev et al., 2000). Meanwhile AMPAR desensitization and the action of different ligands, e.g., kainate, are affected by neuronal transmembrane regulatory proteins (TARPs) (Hansen et al., 2021). Additionally, hippocampal pyramidal neurons express TARP γ-8, which controls the number of AMPA receptors (Rouach et al., 2005). As a possible consequence, these cells produce reasonable amount of glutamate-induced current, in contrast to giant striatal interneurons, whose CP-AMPARs are poorly activated by glutamate (Evlanenkov et al., 2023). Taking into account that effects of AMPAR ligands could depend on the agonist type, we studied the effects of amidine-containing compounds on glutamate-induced responses on CI-AMPARs of hippocampal pyramidal neurons.

CI-AMPARs were activated by glutamate (1 mM) in the presence of D-AP5 (100 µM) to fully exclude NMDA receptors activation. The responses to glutamate exhibited pronounced peak and steady-state components, both of which were completely inhibited by 50 µM DNQX at −80 mV and −20 mV holding potentials (data not shown), indicating that these responses were solely mediated by AMPA receptors.

**FIGURE 5:**
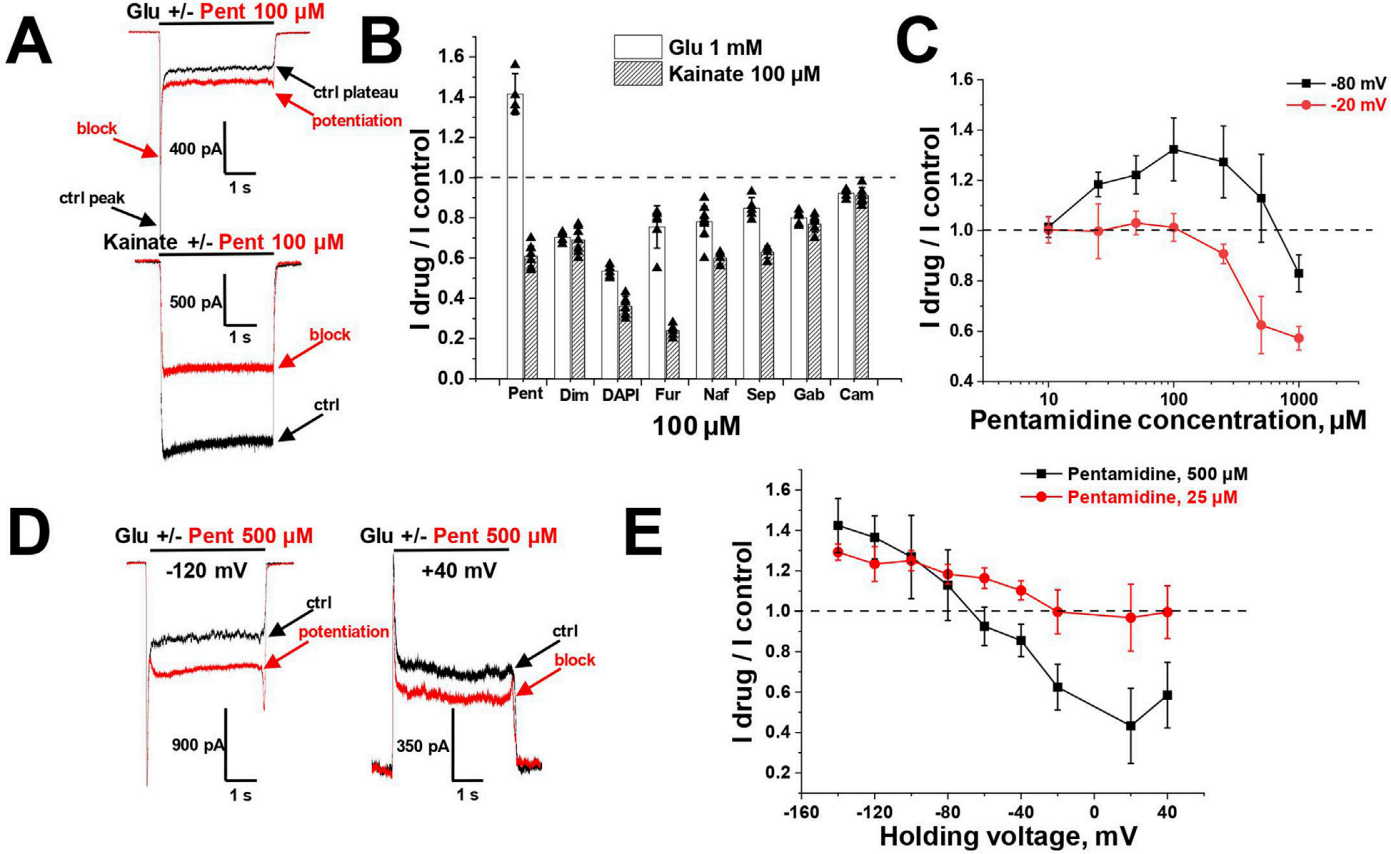
Potentiation and block of glutamate-induced CI-AMPAR currents by pentamidine. **(A)** Representative examples of pentamidine (100 µM) action on glutamate and kainate responses. **(B)** Comparison of amidine-containing compounds action (100 µM) on glutamate (1 mM) and kainate (100 µM) steady-state responses. Pentamidine potentiated glutamate currents and blocked kainate currents, while other compounds blocked both glutamate and kainate currents. **(C)** Concentration dependencies of pentamidine action on glutamate steady-state responses at −80 and −20 mV holding voltages. **(D)** Representative examples of 500 µM pentamidine action at different holding voltages. **(E)** Voltage dependence of 500 and 25 µM pentamidine action on glutamate steady-state responses.

We tested all amidine-containing compounds at a concentration of 100 µM on glutamate-induced steady-state responses at a holding voltage of −80 mV (Figures 5A, B). Diminazene, gabexate, and camostat showed similar inhibition of kainate- and glutamate-induced responses. On the other hand, DAPI, furamidine, nafamostat, and sepimostat were less effective on glutamate-induced steady-state responses compared to kainate-induced ones. It was surprising that pentamidine had a potentiating effect on glutamate-induced steady-state currents (I_drug_/I_control_ = 1.4 ± 0.1, n = 4), suggesting the presence of an additional potentiating mechanism in pentamidine action.

The unusual effect of pentamidine on steady-state glutamate currents was studied in more detail. We examined the concentration-dependencies at holding voltages of −80 and −20 mV (Figure 5C). At −80 mV holding voltage, the potentiating effect increased with an increase in pentamidine concentration, reaching a maximum at 100 µM and decreasing at higher concentrations. The effect transitioned to inhibition at the concentration of 1,000 µM. At −20 mV, low concentrations were ineffective, while at concentrations higher than 100 μM, a blocking effect was observed. Given the voltage-dependent nature of the ratio between potentiation and block, we tested pentamidine action at holding voltages ranging from −140 to +40 mV. We used pentamidine concentrations of 500 and 25 μM, which elicited similar potentiation at −80 mV (Figures 5D, E). At the concentration of 500 μM, pentamidine induced potentiation at hyperpolarized voltages and inhibition at depolarized and positive voltages, with maximal inhibition observed at +20 mV. In contrast, at the concentration of 25 μM, the inhibition at depolarized voltages was less pronounced. These findings can be explained by a combination of voltage-dependent block and voltage-independent potentiation. Potentiation requires lower concentrations than block and is particularly noticeable for 25 µM pentamidine at hyperpolarized voltages, where the voltage-dependent block is minimal. Depolarization and increase in pentamidine concentration led to a predominance of the blocking effect. An important aspect of pentamidine action on glutamate-induced currents is that the peak component of the response was consistently slightly inhibited (10%–30%) regardless of the effect on the steady-state component of the response (Figures 5A–D).

**FIGURE 6:**
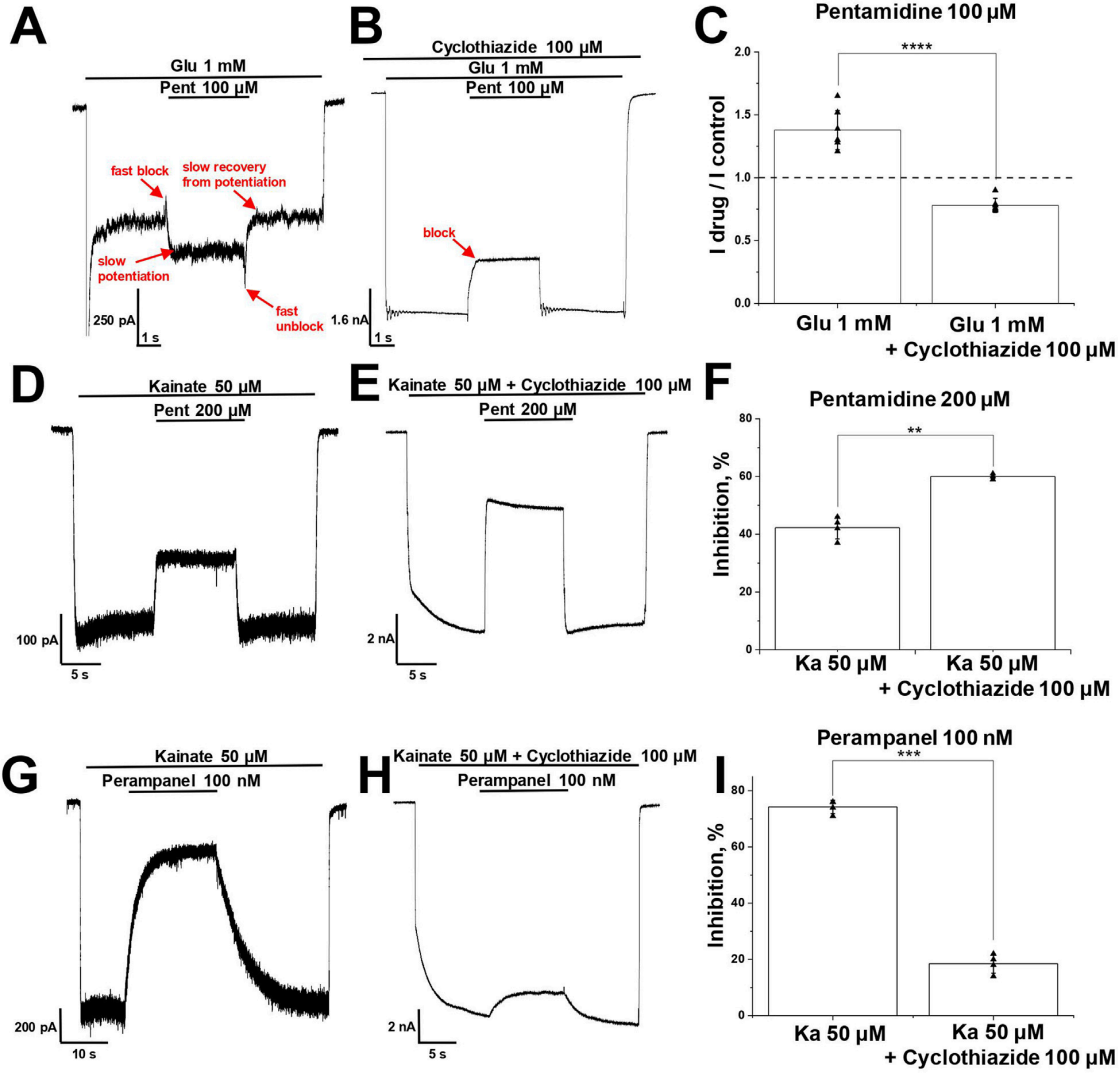
Interaction of pentamidine and perampanel with cyclothiazide on CI-AMPARs. **(A–C)** Potentiation of glutamate-induced CI-AMPAR currents by pentamidine turns to inhibition in presence of cyclothiazide. **(A, B)** Representative examples of pentamidine (100 µM) action in absence **(A)** and presence **(B)** of cyclothiazide (100 μM). **(C)** Summary of data on action of pentamidine on glutamate-induced responses in absence or presence of cyclothiazide. Paired t-test: **** – *p* = 0.00009 (n = 7). **(D–F)** Kainate-induced CI-AMPAR currents inhibition by pentamidine is enhanced in the presence of cyclothiazide. **(D, E)** Representative examples of kainate-induced currents inhibition by 200 µM pentamidine in the absence **(D)** or presence **(E)** of 100 µM cyclothiazide. **(F)** Summary of data on action of pentamidine in the absence or presence of cyclothiazide. Paired t-test: ** – *p* = 0.002 (n = 4). **(G–I)** Kainate-induced CI-AMPAR currents inhibition by perampanel is reduced in the presence of cyclothiazide. **(G, H)** Representative examples of kainate-induced currents inhibition by 100 nM perampanel in the absence **(G)** or presence **(H)** of cyclothiazide. **(I)** Summary of data on action of perampanel in the absence or presence of cyclothiazide. Paired t-test: *** – *p* = 0.0003 (n = 4).

Additionally, in the presence of pentamidine, the steady-state response did not exhibit a monotonic decay from the peak component; rather, the currents reached a minimum followed by a gradual increase (see Figure 5A). The inhibition of the peak component and the slow rise of the steady-state component imply that pentamidine potentiating effect displays slow kinetics compared to its fast blocking effect. This complex action of pentamidine, characterized by potentiating and blocking components with differing kinetics, was also evident in a protocol where pentamidine was applied and removed during glutamate application (Figure 6A). Both the onset of the effect and the recovery were biphasic, with the blocking effect being much faster than the potentiating effect.

### 3.6 Interaction of pentamidine with cyclothiazide

Potentiation of AMPARs is described for a family of compounds called positive allosteric modulators (Frydenvang et al., 2022). The classical compound cyclothiazide causes subtle fast inhibition and strong slow potentiation of AMPARs, which is due to a significant reduction of receptor desensitization (Partin et al., 1993; Patneau et al., 1993). Since desensitization to glutamate is stronger than to kainate, cyclothiazide is significantly more active on glutamate-induced responses than on kainate-induced responses (Vorobjev et al., 2000). If we suppose that pentamidine has a cyclothiazide-like effect, it becomes clear why potentiation was seen for glutamate-induced responses but not for kainate-induced ones. Indeed, in our experiments, cyclothiazide (100 µM) enhanced glutamate-induced currents by 12 ± 3 times (n = 4), whereas potentiation of responses evoked by 100 µM kainate was only 6 ± 3 times (n = 4). If pentamidine causes a cyclothiazide-like effect, the potentiating action on glutamate-induced currents should not be seen in the presence of cyclothiazide. Pentamidine (100 µM) demonstrated 1.4 ± 0.2 times potentiation of steady-state response (Figure 6A), but in the presence of cyclothiazide (100 µM), its effect turned to inhibition (Figure 6B), the I_drug_/I_control_ ratio was 0.8 ± 0.1 (*p* = 0.00009, paired t-test, n = 7, Figure 6C).

In the case of kainate-induced currents, the potentiating effect of pentamidine can be masked by inhibition. To reveal the possible potentiating effect of pentamidine, we compared its action (200 µM) at a relatively low kainate concentration (50 µM) in the absence and presence of a saturating concentration of cyclothiazide (100 µM), which caused an 8 ± 2-fold increase in the current. As expected, pentamidine demonstrated weaker inhibition in the absence (42% ± 4%, Figure 6D) than in the presence (Figure 6E) of cyclothiazide (60% ± 1%, *p* = 0.002, paired t-test, n = 4, Figure 6F). Thus, we can see both the potentiation and inhibition components of pentamidine action on glutamate- and kainate-induced currents. In the case of glutamate-induced currents, potentiation dominates, and we observe an increase in the steady-state response in the presence of pentamidine. In the presence of cyclothiazide, the blocking effect is unmasked. In the case of kainate-induced currents, the blocking effect initially dominates but is partially compensated by potentiating component of action. This compensation is reduced in the presence of cyclothiazide, resulting in a higher level of block. Thus, the presence of cyclothiazide turned the potentiating effect of pentamidine on glutamate-induced current into inhibition and enhanced the inhibition of kainate-induced current. The data support our suggestion that pentamidine has a cyclothiazide-like potentiation effect. However, exact mechanism of potentiation by pentamidine remains unknown.

### 3.7 Pentamidine inhibits the perampanel binding by prevention of channel desensitization

The experiments described above were performed on CI-AMPARs. As glutamate poorly activates CP-AMPARs of giant striatal interneurons, it was impossible to check if pentamidine is able to potentiate glutamate-induced currents in this type of cells. It was also impossible to use cyclothiazide to check if the potentiating action compensates for the block of kainate-induced currents, as we did for CI-AMPARs, because cyclothiazide also weakly affects CP-AMPARs of giant striatal interneurons (Buldakova et al., 2000). To find out if pentamidine could affect channel desensitization on CP-AMPARs, we used an alternative approach.

It is known that the allosteric antagonist perampanel inhibits AMPA receptors by binding to its site in the ion channel collar and decoupling the ligand-binding domains from the ion channel when the channel is closed, stabilizing this state and disrupting channel opening in response to agonist binding (Balannik et al., 2005; Yelshanskaya et al., 2016; Hale et al., 2024). Cyclothiazide prevents AMPA receptor desensitization and stabilizes its open state. The stabilization of opposite channel states by cyclothiazide and perampanel results in the reduction of perampanel activity in the presence of cyclothiazide (Barygin, 2016). Indeed, in experiments with CI-AMPARs, 100 nM perampanel strongly inhibited AMPAR currents in the absence of cyclothiazide (74% ± 2%, Figure 6G) and was practically ineffective in the presence of cyclothiazide (19% ± 3%, *p* = 0.0003, paired t-test, n = 4, Figure 6H), which is in good agreement with our previous results (Barygin, 2016; Dron et al., 2021). A summary of the data is represented in Figure 6I. If there is a cyclothiazide-like effect in the action of pentamidine, we can expect that the activity of perampanel would also be reduced in the presence of pentamidine.

**FIGURE 7:**
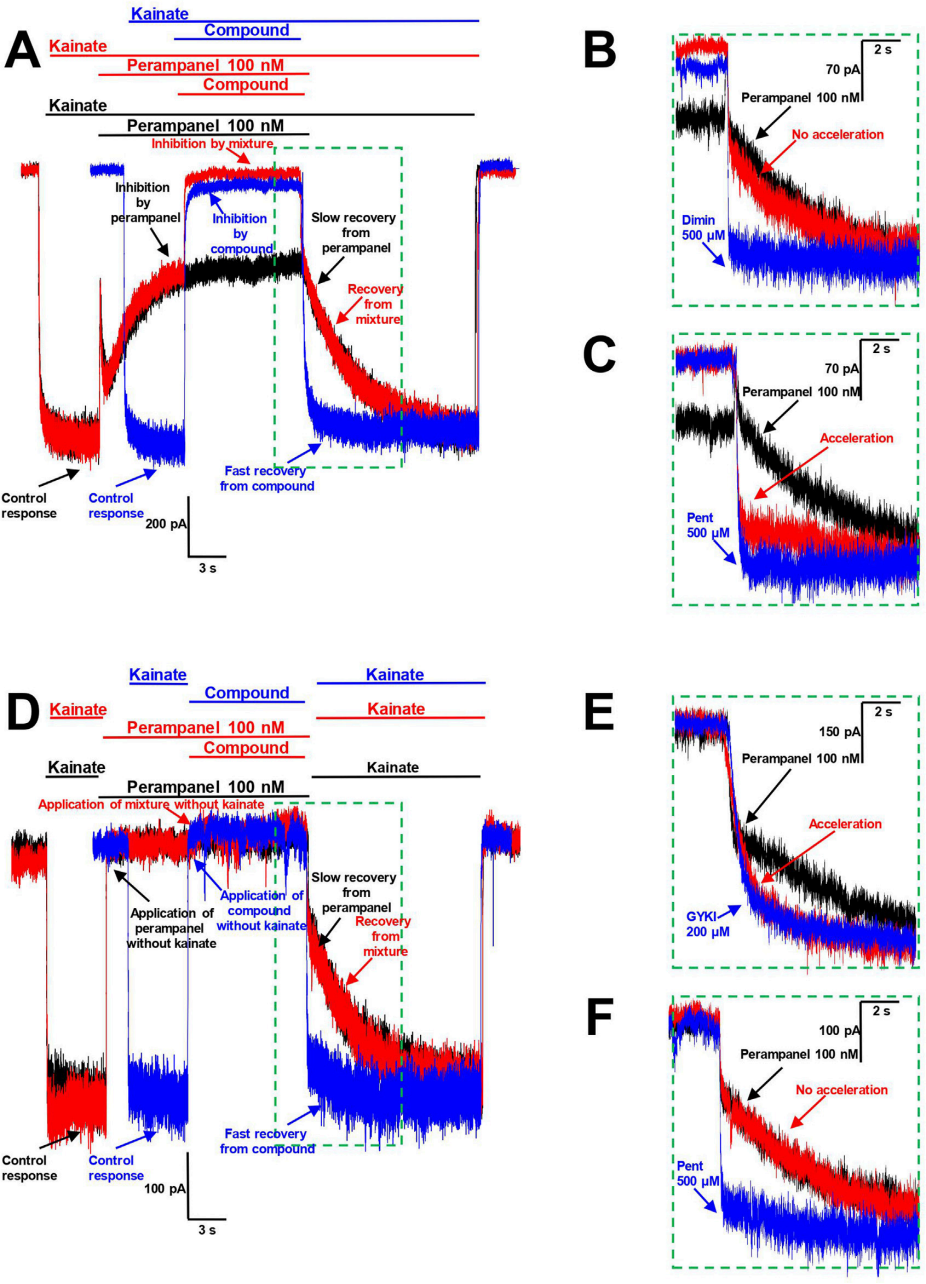
Interaction of pentamidine with perampanel on CP-AMPARs. **(A–C)** Pentamidine inhibits the action of perampanel in presence of agonist. **(A)** The representation of protocols used. Black trace represents the binding of 100 nM perampanel in presence of kainate and its slow washout. Blue trace represents the binding of high concentration of compound tested and its fast washout (IEM-1755 500 µM is shown). Red trace represents the binding of perampanel with sequential binding of compound and recovery from perampanel and compound mixture. **(B, C)** The representation of recoveries in more detail. Diminazene 500 µM **(B)** causes no effect on recovery from perampanel, while pentamidine 500 µM **(C)** causes its significant acceleration. **(D–F)** Pentamidine does not compete with perampanel for the same binding site in absence of agonist. **(D)** The representation of protocols used. Black trace represents the binding of 100 nM perampanel in absence of kainate and its slow washout in presence of kainate. Blue trace represents the application of high concentration of compound tested (pentamidine 500 µM is shown) in absence of kainate and subsequent fast kainate response. Red trace represents the binding of perampanel with sequential application of compound in absence of kainate and recovery from perampanel and compound mixture in presence of kainate. **(E, F)** The representation of recoveries in more detail. GYKI-52466 200 µM **(E)** causes significant acceleration of recovery from perampanel, suggesting the competition for the same binding site, while pentamidine 500 µM **(F)** causes no effect on perampanel washout, suggesting the absence of competition for the same binding site.

The analysis of the interrelation between two compounds, both of which produce inhibition, is a non-trivial task. Therefore, we used a protocol that allows the estimation of relations between two inhibitors with fast and slow kinetics. The washout kinetics of perampanel on CP-AMPARs was slow (τ = 3.6 ± 0.5 s, n = 4), while that for pentamidine (τ = 120 ± 60 ms, n = 4) and for other amidine-containing compounds (except sepimostat) was fast. This difference allowed us to check the possibility of compounds influencing perampanel action by analyzing the washout kinetics in the following protocols (Figure 7A). First, we independently applied a moderate concentration of perampanel (100 nM, 50%–70% inhibition) and a high concentration of the compound tested (90%–100% block) in the presence of kainate (Figure 7A, black and blue traces, respectively). Next, we applied perampanel again, and after achieving inhibition, we applied the mixture of perampanel and the compound tested (Figure 7A, red trace). If the compound with fast washout kinetics prevents the action of perampanel, an acceleration of mixture recovery in comparison with perampanel recovery should be observed. If the effects are independent, the recovery should remain slow.

To test this protocol, we used IEM-1755 (500 µM), a classical blocker of CP-AMPAR channels with fast washout kinetics (Figure 7A). The recovery from the IEM-1755 and perampanel mixture was the same as from perampanel alone, indicating that IEM-1755 has no effect on perampanel binding. This aligns well with the concept that pore blockers are not able to influence the action of perampanel.

In the next stage, we tested amidine-containing compounds with fast washout kinetics in this protocol. Diminazene (500 µM), DAPI (300 µM), furamidine (300 µM), and nafamostat (300 µM) did not accelerate the recovery from perampanel (an example for diminazene is shown in Figure 7B). In contrast, the washout kinetics of the perampanel and pentamidine (500 µM) mixture was significantly accelerated (τ = 620 ± 470 ms, n = 4) compared to that of perampanel alone (τ = 3.6 ± 0.5 s, n = 4), suggesting that pentamidine inhibits the action of perampanel (Figure 7C).

This protocol does not discriminate between competition for the same binding site and allosteric effects. For instance, if pentamidine causes a cyclothiazide-like effect, it can prevent the binding of perampanel by reducing desensitization. To distinguish between these possibilities, we conducted an additional experiment using the same protocol but applying the compounds in the absence of agonist. Since perampanel readily binds to closed AMPARs (Yelshanskaya et al., 2016), testing kainate application results in a slow onset that reflects perampanel washout (Figure 7D, black trace). Desensitization requires agonist binding, so the prevention of channel desensitization could not be observed in the absence of agonist. Therefore, in this case, the acceleration of the response to testing kainate application (Figure 7D, red trace) would indicate competition for the same binding site.

To test this approach, we used GYKI-52466 (200 µM), which is known to exhibit fast washout kinetics and to compete with perampanel for the same binding site (Barygin, 2016) (Figure 7E). The recovery from the perampanel and GYKI-52466 mixture was significantly accelerated compared to perampanel alone, demonstrating that this protocol can clearly identify the competition of the tested compound with perampanel for the same binding site in the closed channel. In contrast to the effect of GYKI-52466, the development of the kainate response after the application of perampanel and pentamidine mixture was as slow as after perampanel alone (Figure 7F), indicating the lack of competition between pentamidine and perampanel for the same binding site.

The significant difference between the results shown in Figures 7C, F clearly demonstrates that pentamidine inhibits perampanel action only when applied in the presence of an agonist. This argues against the possibility of direct competition and supports the suggestion that pentamidine prevents perampanel binding due to reduction of receptor desensitization. However, it is impossible to exclude the possibility that this reduction is the consequence of channel closure prevention.

Taken together, our results demonstrate that in addition to the pore-blocking mechanism, which is manifested for various amidine-containing compounds, pentamidine causes a cyclothiazide-like potentiation effect. Usually, the blocking effect dominates, and the total action is inhibitory. To observe the total effect as potentiation, several factors should be present: (i) CI-AMPA receptors, for which the pore block by organic cations is weak, (ii) hyperpolarized voltages, which additionally reduce the pore block due to permeation through the pore, (iii) activation by glutamate, which causes strong desensitization and enhances the cyclothiazide-like effects, and (iv) a low concentration of pentamidine, which provides stronger binding to the allosteric site than to the channel pore. In other situations, the cyclothiazide-like effect of pentamidine causes attenuation of the total inhibitory effect and can be revealed only by indirect approaches, for instance, by the influence on perampanel action.

Our finding of cyclothiazide-like effect of pentamidine does not necessarily mean binding of both drugs to the same or overlapping binding site. In the absence of high-resolution 3D structure of the AMPA receptor complex with pentamidine, solid data on the binding site may come from intensive mutagenesis studies or from competition experiments, e.g., concentration dependencies for cyclothiazide at different concentrations of pentamidine. Unfortunately, complex action of pentamidine, which also causes activity-dependent pore block, prevents such analysis. Cyclothiazide in the saturating concentration 200 µM caused two times weaker potentiation in the presence of 100 µM pentamidine than in control (data not shown). Thus, we are unable to reach a decisive conclusion on the pentamidine binding site.

### 3.8 Molecular modeling

**FIGURE 8:**
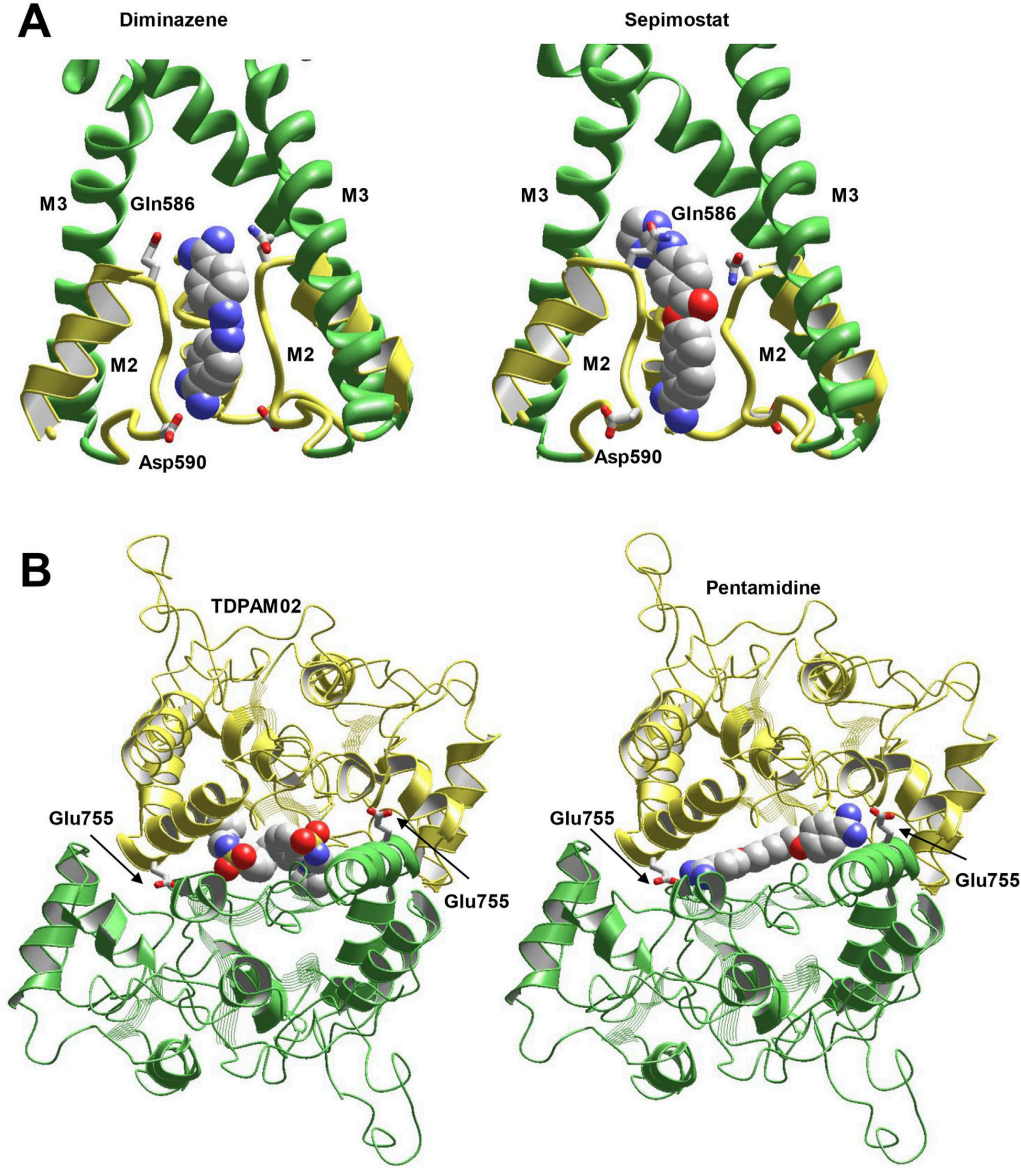
Docking of compounds into the pore **(A)** and cyclothiazide binding site **(B)**. **(A)** Both diminazene (left) and sepimostat (right) bind in the selectivity filter of CP-AMPAR (6dm0 structure) between Gln586 and Asp590. In the optimal binding mode, sepimostat interacts with Asp590 by deeply located charged group, whereas the uncharged end of the molecule remains in the outer vestibule. **(B)** Optimal binding mode of pentamidine (right) in the cyclothiazide site (6hca structure) resembles the binding mode of class III allosteric modulator (left). In addition, amidine group of pentamidine strongly interacts with Glu755 residue.

Nowadays, atomic-scale structures of AMPARs complexed with ligands of various types are available. This allows to rationalize the experimental data in structural terms with a strong background. The 6dm0 structure shows classical pore blocker IEM-1460 (Twomey et al., 2018) in the pore of CP-AMPAR. In agreement with earlier theoretical predictions (Tikhonov et al., 2002), the compound binds at the selectivity filter (Q/R) site in the axial pose. The hydrophobic headgroup remains in the outer vestibule of the open channel, whereas the dicationic tail permeates into the selectivity filter. We performed MCM docking procedure for the compounds studied. Asymmetric compounds were docked in two alternative orientations. The optimal binding modes were found in the selectivity filter region between Gln586 and Asp590, which is in good agreement with experimentally shown binding mode of IEM-1460. Binding modes of diminazene and sepimostat are shown in Figure 8A. Interactions of sepimostat with the channel were stronger if the molecule bound with the charged group located deeply and interacted with Asp590. This preferable binding orientation agrees with experimentally demonstrated large δ_b_ value for this compound.

The chemical structure of pentamidine is quite different from cyclothiazide. However, cyclothiazide belongs to large and structurally diverse family of positive allosteric modulators of AMPA receptors (Frydenvang et al., 2022). Among them, some class III modulators have certain structural similarity with pentamidine. These compounds occupy both binding cyclothiazide sites and therefore bind with one molecule only. We selected the structure 6hca (Laulumaa et al., 2019) that contains bound TDPAM02 molecule. Docking of pentamidine has demonstrated that the molecule readily fits the binding site in the subunit interface. High flexibility of central pentamethylene chain allows pentamidine to bind tightly with numerous residues. The overall location and binding pose match the binding of TDPAM02 and other class III positive allosteric modulators (Figure 8B). In addition, amidine groups of the molecule strongly interact with Glu755 residues, which can serve as critical binding determinants. Although the results of *in silico* docking cannot serve as decisive evidence, they demonstrate a possibility of pentamidine to bind in the cyclothiazide site. Of course, other possible sites and mechanisms of positive allosteric AMPAR modulation by pentamidine should be considered. Also, limited precision of calculations does not allow a conclusion on the effective concentrations for pore block and allosteric potentiation.

## 4 Discussion

**FIGURE 9:**
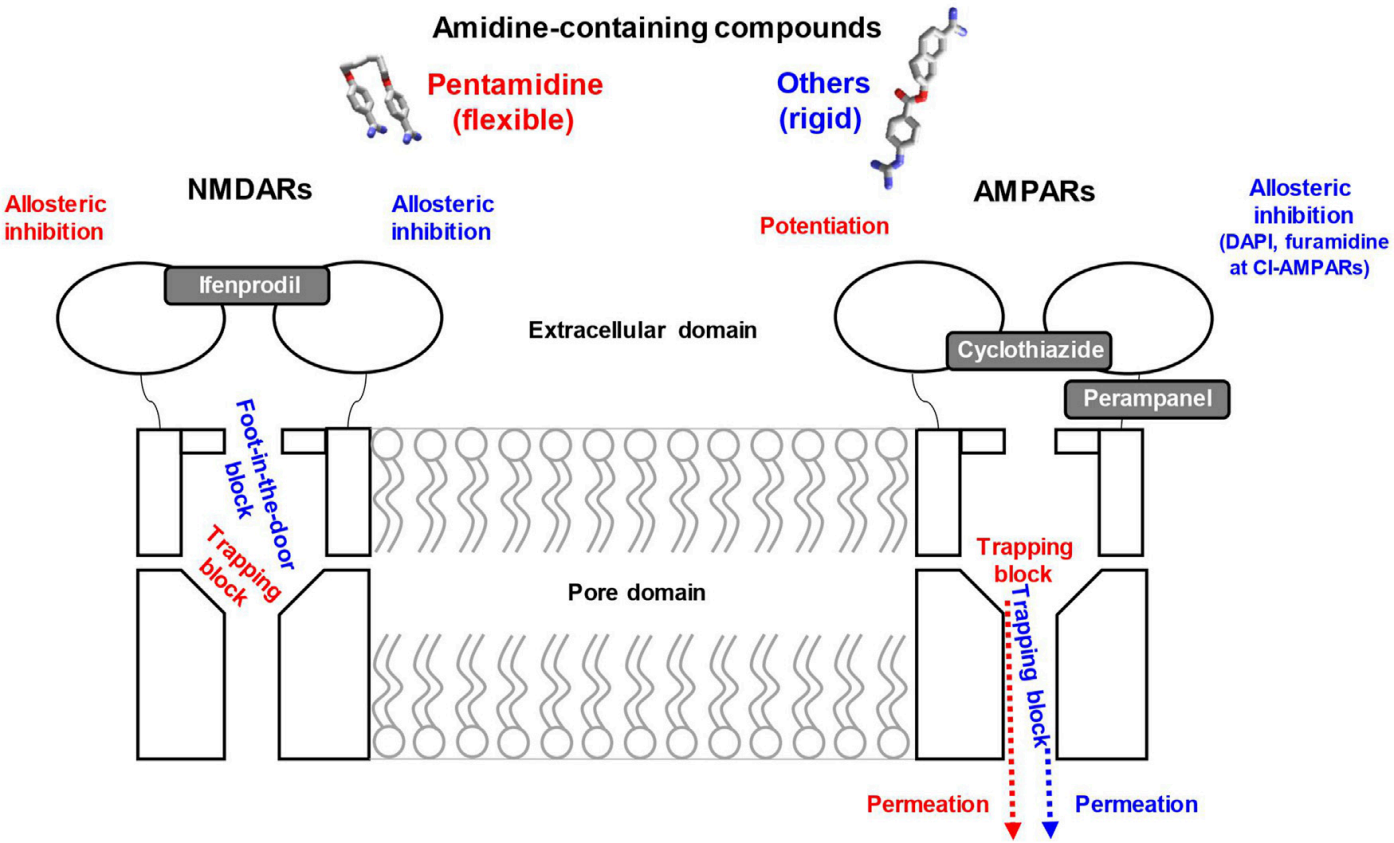
Summary of pentamidine and other amidine-containing compounds effects on NMDA and AMPA receptors. Only flexible pentamidine (red) demonstrates trapping block on NMDA receptors and both block and potentiation on AMPA receptors. Other amidine-containing compounds with rigid structures (blue) combine “foot-in-the-door” block and allosteric inhibition on NMDA receptors and are predominantly permeable trapping blockers of AMPA receptors. Binding site locations in the channel pore are shown in accordance with results, while allosteric binding sites are unknown. Gray symbols schematically indicate binding sites locations of known allosteric modulators.

In this paper, we studied the action of a series of amidine-containing compounds on CP- and CI-AMPARs. Comparisons of the results with the previously studied action on NMDARs (Zhigulin and Barygin, 2023) are shown in Figure 9 and Supplementary Table S3. All compounds except camostat cause strong inhibition of NMDA receptors with IC_50_ values ranging from 0.2 to 16 µM. Additionally, all compounds except camostat and gabexate cause inhibition of CP-AMPA receptors with IC_50_ values of 30–60 µM. Only furamidine and DAPI demonstrated significant inhibition of CI-AMPARs. Therefore, the general tendency of IC_50_ values is NMDAR < CP-AMPAR < CI-AMPAR, although the differences in activity vary significantly. Monocationic compounds gabexate and camostat were not active against both types of AMPA receptors, which is in agreement with data for previously studied compounds with a single charged group (Bolshakov et al., 2005). However, sepimostat, which is also single-charged at physiological pH, demonstrated relatively high activity on CP-AMPARs, which was unusual for such compounds.

Such complex structure-activity relationships suggest a non-uniform mechanism of action. For all active compounds, we revealed a voltage-dependent pore-blocking mechanism of action. In addition, some compounds demonstrated a pronounced voltage-independent component of inhibition on NMDARs and CI-AMPARs. Analysis of the voltage-dependent component demonstrated that it agrees with numerous previous results with various pore blockers of these channels (Bolshakov et al., 2003; Tikhonova et al., 2008; Barygin et al., 2010). In particular, the pore blockers are unable to permeate the NMDAR channel, and their action monotonically increases with hyperpolarization. Computer modeling suggests their binding in the outer vestibule just above the selectivity filter (Dron et al., 2020). In this mode, all compounds except the highly flexible pentamidine act as “foot-in-the-door” blockers. In contrast, the compounds can enter and permeate the relatively wide selectivity filter of AMPARs. As a result, they bind in the deep mode, where they are readily trapped. The membrane electric field can force the compounds to permeate the channel, causing block attenuation at high negative voltages.

Interestingly, potent action on the pore-blocking site (K_b_ value in Supplementary Table S3) in NMDA receptors was found only for furamidine, pentamidine, and nafamostat. Strong inhibition of NMDARs by diminazene, DAPI, sepimostat, and gabexate was due to the voltage-independent component of action (Zhigulin and Barygin, 2023). Most compounds were significantly less active against calcium-impermeable AMPA receptors than against calcium-permeable ones, which is in good agreement with previous studies of AMPA receptor blockers that bind in the Q/R site region (Magazanik et al., 1997). Furamidine and DAPI were rather active against calcium-impermeable AMPA receptors at −80 mV holding voltage, while the activity of other compounds on CI-AMPARs was weak (IC_50_ > 100 µM). This activity was determined by the voltage-independent component of action. The existence of additional binding sites responsible for voltage-independent inhibition of NMDARs and AMPARs was demonstrated in our previous papers (Barygin et al., 2010; Zhigulin and Barygin, 2022; 2023). However, the locations of these sites remain unknown and require further investigations.

It should be noted that the activities of ion channel ligands strongly depend on experimental conditions, which often differ significantly from physiological conditions. The dependence of blocking action on membrane voltage has been discussed above. Another important factor that affects the inhibition of NMDARs is the presence of Mg^2+^ in the physiological environment (Mayer et al., 1984; Nowak et al., 1984). Mg^2+^ ions compete with NMDAR pore blockers and decrease their activities in a voltage-dependent manner (Sobolevsky et al., 1998; Nikolaev et al., 2012). Action of pore blockers may also demonstrate different types of activity-dependence. For NMDARs different interaction of pore-bound drugs with activation and desensitization gates may result in opposite dependence of activity on channel activation (Sobolevsky et al., 1999). For the trapping block of AMPARs the activity-dependence is due to escape of the bound drug molecules into the cytoplasm (Zaitsev et al., 2011). Such factors complicate prediction of drug action in physiological conditions from results of limited *in vitro* experiments.

In order to evaluate the activities of amidine-containing compounds under conditions more representative of physiological situation, we conducted a comparison of their effects on AMPARs activated by the endogenous neurotransmitter glutamate. The result was somewhat surprising. While the majority of compounds inhibited glutamate-activated steady-state currents as well as kainate-activated currents, pentamidine caused significant potentiation of glutamate-induced steady-state responses under experimental conditions that are unfavorable for pore block. This effect cannot be explained by pore block mechanism. Even if the pore bound pentamidine prevents AMPAR desensitization, the pore remains blocked and effect cannot reverse from inhibition to potentiation.

A more detailed analysis has shown that the potentiating effect is similar to the action of cyclothiazide. In the case of kainate-induced currents, this type of action is masked by the pore block because the cyclothiazide-like anti-desensitizing effects are less pronounced for kainate-induced currents with a moderate level of desensitization compared to deeply-desensitizing glutamate-induced currents.

The very specific action of pentamidine can be explained by the flexibility of this molecule, which, unlike other compounds in this study, can adopt numerous bent and folded conformations (Dron et al., 2020). While the similarity between pentamidine and cyclothiazide is relatively low, some structures of positive allosteric AMPAR modulators (Frydenvang et al., 2022) bear a resemblance to pentamidine. The possibility of pentamidine to bind at the site targeted by positive allosteric AMPAR modulators was supported by docking calculations. However, it should be spelled out that bold evidences of pentamidine binding to the cyclothiazide site are absent and this issue requires further investigations. Since neuronal AMPA receptors include auxiliary proteins in addition to the GluA subunits that form their ion channel (Hansen et al., 2021), the potentiation by pentamidine could as well be due to an interaction with a TARP, or cornichon.

In the present study, we systematically analyzed the effects of a series of amidine-containing compounds on CI- and CP-AMPARs. This work, along with our previous research on NMDARs (Zhigulin and Barygin, 2023), offers a comprehensive overview of this novel group of ionotropic glutamate receptor inhibitors with a complex pharmacological profile. We observed that despite their overall structural similarity, these compounds exhibit a remarkable diversity of effects and mechanisms of action. Therefore, the structure-function relationships within this group are highly intricate and strongly influenced by experimental conditions. This highlights the significance of performing a comparative analysis of compound interactions with various types of glutamate receptors and providing detailed characterizations of each compound effects during the development of new pharmaceuticals.

## Data Availability

The raw data supporting the conclusions of this article will be made available by the authors, without undue reservation.

## Abbreviations

AMPA, α-amino-3-hydroxy-5-methyl-4-isoxazolepropionic acid; CI-AMPARs, calcium-impermeable AMPA receptors; CP-AMPARs, calcium-permeable AMPA receptors; NMDARs, N-methyl-D-aspartate receptors.
