# Supplementary material for: The diversity of AMPA receptor inhibition mechanisms among amidine-containing compounds
Source: Front Pharmacol. 2024 Oct 9;15:1467266. doi: 10.3389/fphar.2024.1467266 (PMC11496081; doi:10.3389/fphar.2024.1467266)
Supplement: Supplementary file 1 [file Table1.DOCX]

Supplementary Material

# Supplementary Tables

Table S1. Activity of amidine-containing compounds against CP- and CI-AMPARs.

| Compound | Chemical structure | Block at 100 µM, % | | IC_50_, µM | |
| --- | --- | --- | --- | --- | --- |
|  |  | CP-AMPARs | CI-AMPARs | CP-AMPARs | CI-AMPARs |
| Pentamidine |  | 70 ± 4 (n=4) | 39 ± 5 (n=8) | 43 ± 9 (n=4) | >100 |
| Diminazene |  | 69 ± 4 * | 31 ± 7 * | 60 ± 11 * | >100 * |
| DAPI |  | 83 ± 4 (n=4) | 64 ± 5 (n=6) | 27 ± 1 (n=4) | 59 ± 9 (n=6) |
| Furamidine |  | 79 ± 7 (n=4) | 76 ± 3 (n=7) | 38 ± 11 (n=4) | 47 ± 8 (n=7) |
| Nafamostat |  | 60 ± 11 (n=4) | 40 ± 3 (n=4) | 55 ± 24 (n=4) | >100 |
| Sepimostat |  | 66 ± 5 (n=4) | 37 ± 3 (n=5) | 56 ± 6 (n=4) | >100 |
| Gabexate |  | 30 ± 4 (n=4) | 23 ± 4 (n=6) | >100 | >100 |
| Camostat |  | 16 ± 4 (n=4) | 9 ± 4 (n=6) | >100 | >100 |

* the data from ([Zhigulin et al., 2022](#_ENREF_82))

Table S2. The δ_b_ values and constants of binding to channel (K_b_) and superficial (K_vin_) sites obtained from fitting by eq. 1 (CP-AMPARs) and eq. 2 (CI-AMPARs) for amidine-containing compounds

| Compound | Concentration used, µM  CP-AMPARs/  CI-AMPARs | CP-AMPARs | | CI-AMPARs* | |
| --- | --- | --- | --- | --- | --- |
|  |  | δ_b_ | K_b_, µM | K_b_, µM | K_vin_, µM |
| Pentamidine | 100/200 | 0.49 ± 0.04 | 36 ± 4 | 130 ± 30 | 800 ± 200 |
| Diminazene | 60/200 | 0.64 ± 0.05 | 47 ± 5 | 550 ± 250 | 560 ± 110 |
| DAPI | 30/60 | 0.65 ± 0.08 | 5.4 ± 1.2 | 24 ± 7 | 62 ± 6 |
| Furamidine | 50/50 | 0.72 ± 0.15 | 15 ± 5 | 110 ± 70 | 59 ± 6 |
| Nafamostat | 60/200 | 0.76 ± 0.16 | 9 ± 4 | 81 ± 27 | 420 ± 80 |
| Sepimostat | 60/200 | 0.88 ± 0.12 | 71 ± 9 | 180 ± 120 | 530 ± 190 |

The data from different cells (n ≥ 4 for each holding potential) were pooled together and fitted with eq. 1 or 2. Approximation error values were taken as the precision measures; * binding constants calculated using eq. 2 with fixed δ_b_ values, obtained from the CP-AMPARs data.

Table S3. Comparison of NMDA and AMPA receptor inhibition characteristics for amidine-containing compounds

| **Compound** | **IC_50_ at -80mV, μM** | | | **Action at channel site** | | | | | | **Action at superficial site** | |
| --- | --- | --- | --- | --- | --- | --- | --- | --- | --- | --- | --- |
|  |  |  |  | **δ_b_** | | | **K_b_, μM** | | | **K_vin_, μM** | |
|  | **NMDAR** | **CP-AMPAR** | **CI-AMPAR** | **NMDAR** | **CP-AMPAR** | **CI-AMPAR** | **NMDAR** | **CP-AMPAR** | **CI-AMPAR** | **NMDAR** | **CI-AMPAR** |
| Pentamidine | 0.41 | 43 | >100 | 0.7 | 0.5 | 0.5 | 27 | 36 | 130 | 10 | 800 |
| Diminazene | 13 | 60 | >100 | 0.55 | 0.65 | 0.65 | 1500 | 47 | 550 | 14 | 560 |
| DAPI | 3.1 | 27 | 59 | 0.55 | 0.65 | 0.65 | 5000 | 5.4 | 24 | 3.2 | 62 |
| Furamidine | 0.64 | 38 | 47 | 0.55 | 0.7 | 0.7 | 22 | 15 | 110 | 3.3 | 59 |
| Nafamostat | 0.20 | 61 | >100 | 0.55 | 0.75 | 0.75 | 11 | 9 | 81 | 3.0 | 420 |
| Sepimostat | 3.5 | 56 | >100 | 0.55 | 0.9 | 0.9 | 102 | 70 | 180 | 7.0 | 530 |
| Gabexate | 16 | >100 | >100 | 0.55 | n.a.* | n.a. | 800 | n.a. | n.a. | 25 | n.a. |
| Camostat | >100 | >100 | >100 | n.a. | n.a. | n.a. | n.a. | n.a. | n.a. | n.a. | n.a. |

*not applicable
